# Risk factors for the development of macular edema in children with uveitis

**DOI:** 10.3389/fopht.2023.1134077

**Published:** 2023-06-02

**Authors:** Ronit Friling, Ori Berliner, Maya Eiger-Moscovich, Yi-Hsing Chen, Oren Tomkins-Netzer, Michal Kramer

**Affiliations:** ^1^ Unit of Pediatric Ophthalmology, Schneider Children’s Medical Center of Israel, Petach Tikva, Israel; ^2^ Faculty of Medicine, Tel Aviv University, Tel Aviv, Israel; ^3^ Department of Ophthalmology, Rabin Medical Center – Beilinson Hospital, Petach Tikva, Israel; ^4^ Moorfields Eye Hospital, Institute of Ophthalmology, University College London, London, United Kingdom; ^5^ Department of Ophthalmology, Lady Davis Carmel Medical Center, Ruth and Bruce Rappaport Faculty of Medicine, Technion – Israel Institute of Technology, Haifa, Israel

**Keywords:** uveitis, macular edema, children, risk factors, complications

## Abstract

**Aim:**

To determine the risk factors for macular edema (ME) in children with uveitis.

**Methods:**

A retrospective study was conducted of 150 pediatric patients (264 eyes) with uveitis attending 2 tertiary medical centers. Data were collected from the medical files on demographics, type of uveitis, etiology, clinical findings, treatment, and time to development of ME. Risk factors for the development of ME were identified.

**Results:**

ME developed in 63 eyes (23.9%) over a mean period of 15.3 ± 2.95 months from diagnosis of uveitis, at a rate of 0.08 eyes per eye-year. On univariate analysis, risk factors for the development of ME were the non-anterior location of the inflammation (*p*=0.002), band keratopathy (*p <*0.0001), posterior synechiae (*p*=0.003), cataract (*p*=0.002), and vision impairment at presentation (*p <*0.0001). On multivariate analysis, non-anterior uveitis, which includes intermediate, pan, and posterior-uveitis, and vision impairment retained significance as independent risk factors of ME.

**Conclusion:**

Within the pediatric population with uveitis, non-anterior location is associated with the highest risk of ME, followed by the presence of complications, such as band keratopathy and posterior synechiae. These findings indicate a need for close follow-up in children with uveitis for early detection of ME.

## 1 Introduction

Pediatric uveitis poses unique diagnostic and treatment challenges including the difficulty in performing thorough examinations which are necessary to identify and treat potential complications. Common causes of visual impairment in children with uveitis are cataract formation, glaucoma, band keratopathy, and macular edema (ME). They are also at increased risk of developing deep amblyopia (1, 2). The reported prevalence rate of ME in pediatric cohort studies ranges from 17% at presentation up to 30%, depending on the duration of follow-up (3).

Studies of ME in uveitic patients mostly include adult patients. Identified independent risk factors for ME in these patients include the primary non-anterior location of the inflammation (intermediate, posterior, and pan-uveitis), advanced age, active smoking, presence of an epiretinal membrane, and absence of a posterior vitreous detachment (4–6). Studies addressing ME in children are scarce and mostly include smaller cohorts of specific disease entities. The prevalence of ME for pediatric uveitis patients was noted to vary by anatomic location, with similar rates reported in anterior and intermediate uveitis, followed by posterior and pan-uveitis (7–9). Pars planitis was reported by Rosenberg et al. to be associated with a two-fold increased risk of ME compared to juvenile idiopathic arthritis (JIA) (2). The rate of development of ME over time was 0.068 per eye-year in the study of Paroli et al. (8) for intermediate uveitis, while corresponding values in patients with JIA were 0.04 in the SITE study (10). The prevalence of ME in pediatric uveitis increased with a longer duration of disease (3). For intermediate uveitis, an average interval of 5.7 years was reported between the onset of intermediate uveitis and the development of ME (11). Age younger than 7 years was found to be associated with a higher risk of complications, including ME (12).

The aim of the present study was to further investigate a larger cohort of pediatric uveitis patients and to search for possible risk factors for ME. Identifying possible risk factors in this unique group of patients may guide follow-up and imaging required for early diagnosis and treatment of ME.

## 2 Methods

A retrospective cohort study design was used. The electronic databases of a tertiary medical center in Israel and a major specialist eye hospital in the UK were searched for all patients younger than 18 years old who were diagnosed with uveitis in 2005-2018; and 1980-2018, respectively. The study adhered to the tenets of the Declaration of Helsinki and was approved by the local institutional review boards (Rabin Medical Center, 0725-15-RMC, and Moorfields Eye Hospital, Department of Research and Development, ROAD19039).

The following data were retrieved from the patient files: age and sex, age at presentation, type of uveitis based on the Standardization of Uveitis Nomenclature (SUN) classification (1), etiology of uveitis, treatment, development of ME, time to development of ME, complications at presentation and during follow-up, and visual acuity at presentation and during the disease course. The diagnosis of ME was made based on clinical findings during the ophthalmic examination and either intraretinal fluid or cysts and/or subretinal fluid on optical coherence tomography (OCT) or petaloid leakage on fluorescein angiography.

Patients were only included if macular imaging was sufficient to clearly demonstrate the pathology. In addition, Group A only included patients who developed macular edema prior to cataract surgery. Other complications were categorized as posterior synechiae, band keratopathy, elevated intraocular pressure (above 21 mmHg), glaucoma, and cataract. Vision impairment or loss was defined as visual acuity less than 6/12.

Patients followed for less than 6 months were excluded. The remainder were divided into two groups by the presence or absence of ME.

The majority of cases with uveitis involving the posterior segments of the eye, as defined by the SUN classification, had intermediate uveitis, followed by the group of pan- and posterior uveitis (**Table 1**). Therefore, for statistical analysis, intermediate, posterior, and panuveitis were grouped together as non-anterior uveitis.

**Table 1:** Risk factors for macular edema based on clinical characteristics (150 patients, 264 eyes).

	Total n (%)	Macular edema n (%)	No macular edema n (%)	*P* value
Female	83/150 (55.3)	20/36 (55.6)	63/114 (55.3)	1.00
Bilateral uveitis, patients	36/150 (24.0)	8/36 (22.2)	28/114 (24.6)	0.83
Right eye	129/264 (48.9)	30/63 (47.6)	99/201 (49.3)	0.28
Age at presentation, yr (mean ± SEM)	9.05 ± 0.32	8.94 ± 0.63	9.08 ± 0.38	0.86
Non-infectious uveitis	247/264 (93.6)	61/63 (96.8)	186/201 (92.5)	0.11
Uveitis location				0.03
Anterior uveitis	137/264 (51.9)	20/63 (31.7)	117/201 (58.3)	
Intermediate uveitis	93/264 (35.2)	32/63 (50.8)	61/201 (30.3)	
Posterior+panuveitis	34/264 (12.9)	11/63 (17.5)	23/201 (11.4)	
Non-anterior uveitis	127 (48.1)	43/63 (68.3)	84/201 (41.7)	0.007
Findings at presentation				
Cataract	29 (12.2)	14/63 (23.0)	15/201 (8.5)	0.03
Band keratopathy	17 (7.1)	11/63 (18.0)	6/201 (3.4)	0.001
Posterior synechiae	54 (22.8)	23/63 (38.3)	31/201 (17.5)	0.009
Glaucoma	10 (4.2)	4 (6.6)	6/201 (3.4)	0.41
BCVA, LogMAR mean ± SEM	0.32 ± 0.03	0.47 ± 0.05	0.25 ± 0.04	0.002

BCVA, best corrected visual acuity.

### 2.1 Statistical analysis

Patient information was entered into an Excel spreadsheet (version 2017, Microsoft Corp.), and analyses were performed using SPSS statistical software (version 25, IBM). Generalized estimate equations were used to compare characteristics between the groups. Survival to the occurrence of ME was calculated using a Kaplan-Meier estimator. To analyze the relationship between ME and clinical features at presentation, a Cox linear regression model was used; hazard ratios were calculated and correlations were adjusted between the two eyes of the same patient and the variable length of follow-up. For patients who presented with ME, follow-up time was labeled 0. Only factors found to be significant in univariate analysis (crude) were included in the multivariate step (refined). Continuous data are presented as mean ± standard error of the mean and categorical data as proportions. A *p*-value of <0.05 was considered significant.

## 3 Results

The cohort included 150 pediatric patients (264 eyes), 83 females and 67 males, of mean age 9.03 ± 0.25 years at presentation [median 9 years, interquartile range (IQR) 6-12 years, 754.4 eye-years]. Infectious uveitis was diagnosed in 4 patients (4 eyes), 3 with herpes simplex virus and one with toxoplasmosis. The mean follow-up time was 51.95 ± 4.04 months (median 33 months, IQR 17.5-72.5 months).

ME developed in 63 eyes of 36 patients (23.9%) (bilaterally in 27 patients). It was already present at presentation in 21 eyes (8%), including 14 with non-anterior uveitis, and developed during follow-up in the remainder, at a rate of 0.08 per eye-year: 0.05 per eye-year for anterior uveitis and 0.12 per eye year for non-anterior uveitis. The mean time from diagnosis of uveitis to the development of ME was 15.3 ± 2.95 months (median 6 months, IQR 0-21 months). There was no difference between patients in whom ME was present at diagnosis or developed during the disease course in anatomic distribution (33.3% non-anterior *vs*. 31.0% anterior, *p*=1.00) or age at presentation (9.81 ± 0.86 years *vs*. 8.43 ± 0.62 years, *p*=0.2). Information regarding the status of inflammation was available for 44 eyes of group A, of which 39 were active when ME was diagnosed.

**Table 1** compares the background and clinical factors between patients/eyes with and without ME. Of the 63 eyes with ME, 20 (31.7%) had anterior uveitis and 43 (68.3%) had non-anterior uveitis, including 32 (50.8%) with intermediate uveitis and 11 (17.5%) with panuveitis. There was only one case of isolated posterior uveitis without ME. ME occurred more often in eyes with non-anterior compared to anterior uveitis (*p*=0.007).

**Figure 1:**
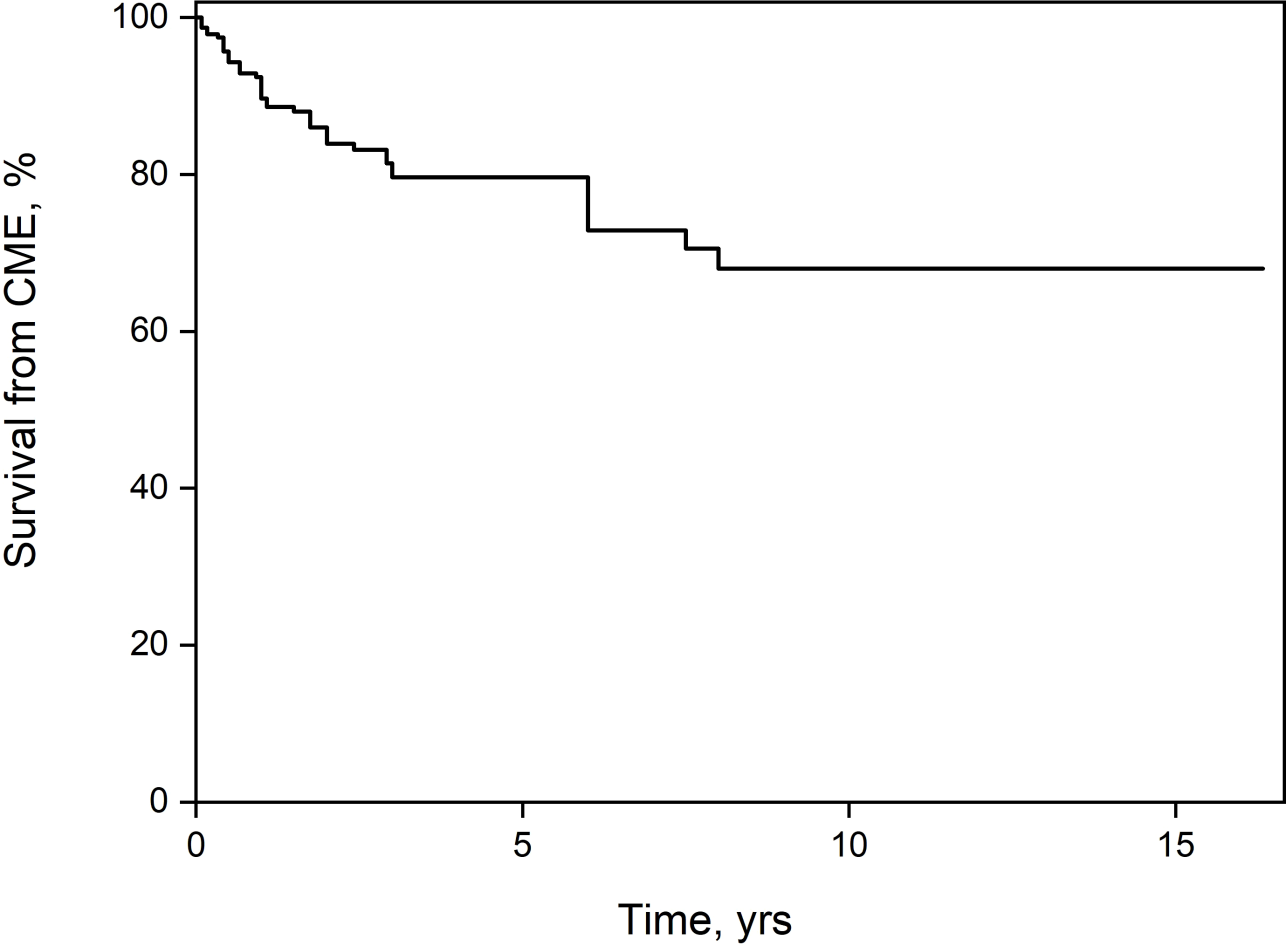
Kaplan-Meier survival estimator. After 10 years of follow-up, 38.64% of the patients had developed macular edema.

Among the patients with anterior uveitis, there was no significant difference in the rate of development of ME between patients with (n=8, 20%) or without (n=12, 12.4%) JIA (*p*=0.29). Additional complications in eyes with ME were cataracts in 14 (23%), band keratopathy in 11 (18%), and posterior synechiae in 23 (38.3%). These rates were significantly higher than in patients without ME. The between-group difference in visual acuity was significant as well (**Table 1**). Only one patient with pars planitis demonstrated ERM. None of the patients developed hypotony. A comparison of the treatment regimens revealed that patients who ultimately developed ME were more likely than patients without ME to receive local corticosteroid injections (14.3% *vs*. 0.9%, *p <*0.0001) and systemic corticosteroids (63.5% *vs*. 40.2%, *p*=0.004), and less likely to receive topical corticosteroids (74.2% *vs*. 89.2%, *p*=0.02). There was no difference between the groups in the use of second-line immunosuppression (41.3% *vs*. 39.3%, *p*=0.87). Disregarding the eyes with ME at presentation (n=20), in the remainder, the percentage of eyes in which ME did not ultimately develop was 84.53% at 1 year after diagnosis of uveitis, 72.33% at 5 years, and 61.36% at 10 years (**Figure 1**).

On univariate analysis of potential risk factors, there was a significant correlation between the development of ME with non-anterior uveitis (HR 2.34, 95% CI 1.37-3.97, *p*=0.002) as well as the presence of posterior synechiae (HR 2.21, 95% CI 1.31-3.73, *p*=0.003), band keratopathy (HR 3.43, 95% CI 1.78-6.61, *p <*0.0001), cataract at presentation (HR 2.63, 95% CI 1.44-4.8, *p*=0.002), and vision loss at presentation (HR 3.02, 95% CI 1.79-5.09, *p <*0.0001). When these parameters were entered into the multivariate model, non-anterior uveitis (HR 2.8, 95% CI 1.6-4.92, *p <*0.0001) band keratopathy (HR 2.51, 95% CI 1.18-5.35, *p*=0.02), and vision loss (HR 2.09, 95% CI 1.21-3.61, *p <*0.01) retained statistical significance as independent risk factors (**Table 1**).

## 4 Discussion

The purpose of the study was to identify risk factors for the development of ME, a leading cause of vision loss in children and adults with uveitis (13–15). In the present pediatric cohort, ME developed in 63 of 264 eyes with uveitis: 31.7% of them in eyes with anterior uveitis, 50.8% of them in eyes with intermediate uveitis, and 17.5% of them in eyes with panuveitis. Non-anterior location of the inflammation was the most significant independent risk factor for ME. However, ME was also associated with complications of the anterior segment, such as band keratopathy, posterior synechiae, and cataracts.

In earlier studies of mostly adult patients, rates of ME associated with non-anterior uveitis ranged from 20% to 30% (16–20). In children, similar to the present study, the prevalence of ME differed by anatomic location: 21% to 44% for anterior uveitis (3%-24% of cases of JIA uveitis) compared to 28%-40% for intermediate uveitis, 14%-17% for posterior uveitis, and 13%-18% for panuveitis (7–9). The rate of development of ME over time in our study was 0.08 per eye-year, similar to the rate of 0.068 per eye-year, reported by Paroli et al. (8); corresponding values in patients with JIA were 0.05 in our study and 0.04 in the SITE study (10). Smith et al. (3) found that the prevalence of ME in pediatric uveitis increased with a longer duration of disease, from 17% of patients at 1 year to 35% at 5 years. Donaldson et al. (11) reported an average interval of 5.7 years between the onset of intermediate uveitis and the development of ME. By contrast, the survival curve in the present study indicated a shorter mean interval of only 15.3 ± 2.95 months (median 12 months, IQR 6-27.8 months). Similar to our results, Rosenberg et al. (2) observed that pars planitis was associated with a higher risk of ME, double that of JIA. Several authors found that potential risk factors, such as gender (2, 3), age of onset, and immunosuppressive treatment (3) had no significant effect on the development of ME in patients with JIA uveitis. However, specifically in patients with intermediate uveitis, age younger than 7 years was associated with a higher risk of complications, including ME (12). Kalinina Ayuso et al. (21) attributed male gender and uveitis as the first manifestation of risk factors for ME in patients with JIA. In the present study, we found no difference in mean age between patients with or without ME.

A possible explanation for the difference in ME rate between anterior and non-anterior uveitis may lie in their different pathophysiologic mechanisms. The process of inflammation-related-ME involves the disruption of the blood-retinal barrier by activated T cells, followed by intra- and extracellular fluid accumulation within the macular retina (13). Breakdown of the blood-retinal barrier is more likely when inflammation involves the posterior segment of the eye. Our finding that all cases of non-anterior uveitis were either intermediate uveitis or panuveitis supports the hypothesis that vitritis plays a role in the development of ME. This hypothesis may gain additional support by the fact that most of the patients who developed ME and for whom data were available were active when ME developed.

This study demonstrated an association between ME and anterior segment complications, such as posterior synechiae, band keratopathy, and cataracts, which are prevalent in JIA-related and idiopathic chronic anterior uveitis. Others found that the presence of posterior synechiae was a poor prognostic factor in JIA-related uveitis, with final vision in affected patients of ≤20/200 (22). Posterior synechiae develop as a result of ongoing inflammation and therefore can serve as an indicator of additional complications, including ME. Severe uveitis at the initial ocular examination, characterized by complications, longer duration, a long time before referral, and younger patient age, has been associated with a worse prognosis (21, 22). Accordingly, in the present study, the between-group differences in treatment patterns before the development of ME suggest that the patients in whom ME developed during follow-up had more aggressive uveitis, requiring systemic corticosteroids and local injections.

In our cohort, the rate of ME in eyes with uveitis was 8% at presentation and increased to 32% over a 10-year follow-up. This finding is in agreement with some earlier studies demonstrating an increased risk of the development of ME and other complications during follow-up in patients with uveitis (3). However, one study of 33 children with pars planitis showed that ME developed in 31%, of whom 67% were diagnosed at presentation and only 33% at a mean of 57 months from presentation (23). The difference among the studies may be attributable to differences in referral practices. Heinz and colleagues (24), in a study of pediatric and adult uveitis, found that about half the eyes of the children had no ocular-related complication at presentation compared to 39% of the adult eyes. The most frequent complication in both groups was ME, followed (in children) by cataracts (24). Hence, in our study, that vision loss at presentation was a risk factor for ME suggests that this factor reflects the severity of the disease and probably the presence of other complications. The diagnosis of ME is challenging in children, and ophthalmologists need to rely on a combination of clinical examination and OCT images (25, 26). It is important that they be alert to risk factors for its development, in order to maintain close follow-up and administer strict long-term treatment regimens to prevent complications and preserve vision.

This study was limited by the retrospective design, particularly the potential selection bias posed by conducting the study in a tertiary center which most likely sees more severe cases. The retrospective design also limited our ability to assess the accumulated time of disease activity and quiescence. The longer inflammation is active, the greater the chance for developing complications, and may therefore serve as a surrogate for activity. Additional selection bias led to the exclusion of children whose cataracts limited posterior segment imaging. At the same time, despite the aforementioned limitations, the study included a large cohort of only pediatric patients with uveitis followed for a long duration and for whom extensive clinical information was available, reflecting the real-life experience in specialized uveitis centers. This allowed us to examine the impact of many factors, highlighting the need for early diagnosis, prompt treatment, and close monitoring to prevent sight-threatening complications in children with non-anterior uveitis and complicated anterior uveitis. Our results provide additional important information that may help clinicians evaluate these children and plan their follow-up.

In childhood uveitis, particularly intermediate uveitis, ME may be present at first evaluation. Therefore, a prospective study with longitudinal follow-up of visual acuity is needed to provide further insight into the mechanisms underlying the process from the onset of ME to the development of visual impairment and appropriate means of monitoring and aggressive treatment.

In conclusion, this study showed that among pediatric patients with uveitis, non-anterior uveitis and structural complications related to severe anterior uveitis are independent risk factors for the development of ME.

## Data availability statement

The raw data supporting the conclusions of this article will be made available by the authors, without undue reservation.

## Ethics statement

The studies involving human participants were reviewed and approved by Rabin Medical Center, 0725-15-RMC, and Moorfields Eye Hospital, Department of Research and Development (ROAD19039). Written informed consent from the patients/participants was not required to participate in this study in accordance with the national legislation and the institutional requirements.

## Author contributions

Conception and design: RF, OT-N, KM. Analysis and interpretation: RF, OB, OT-N,MK. Data collection: OB, ME-M, Y-HC. Overall responsibility: RF, OB, ME-M, Y-HC, OT-N, MK. All authors contributed to the article and approved the submitted version.
